# Rodent indent not self-evident: a case of mistaken identity of the ‘Chicago Rat Hole’

**DOI:** 10.1098/rsbl.2025.0343

**Published:** 2025-10-15

**Authors:** Michael C. Granatosky, Gabby Guilhon, Noah D. Chernik, Stratos J. Kantonis, Christine J. Lee, Edwin Dickinson

**Affiliations:** ^1^ Department of Ecology and Evolutionary Biology, The University of Tennessee Knoxville, Knoxville, TN, USA; ^2^ Duke Lemur Center, Durham, NC, USA; ^3^ Department of Anatomy, New York Institute of Technology College of Osteopathic Medicine, Old Westbury, NY, USA; ^4^ Center for Biomedical Innovation, New York Institute of Technology College of Osteopathic Medicine, Old Westbury, NY, USA; ^5^ Department of Anthroplogy and Archaeology, Calgary AB, Calgary, Canada

**Keywords:** science communication, public science engagement, Rodentia, urban ecology, neoichnology

## Abstract

The ‘Chicago Rat Hole’ is a remarkable full body impression ostensibly created by a brown rat (*Rattus norvegicus*) crossing fresh concrete in Chicago’s Roscoe Village that became a viral sensation. While the public attributed the mark to a brown rat, no formal analysis had been conducted to confirm its identity. Using clear anatomical landmarks, we compared measurements from the ‘Chicago Rat Hole’ to eight sympatric rodent species using univariate and multivariate analyses. Univariate tests showed no significant differences in snout-to-tail length, head width, tail-base width or third digit length between the imprint and members of the genus *Sciurus* (i.e. tree squirrels). Discriminant function analysis indicated a 98.67% likelihood that the ‘Chicago Rat Hole’ was a squirrel, with classifications split between the eastern grey squirrel (50.67%) and the fox squirrel (48.00%). Given local population densities, an eastern grey squirrel likely represents the most parsimonious species-level match. This investigation underscores the challenges of attributing a trace to the tracemaker. While we acknowledge the playful spirit of this investigation, our broader aim is to highlight that scientific inquiry begins with curiosity and observation: qualities that are not exclusive to professional scientists, but accessible to anyone with an interest in understanding the natural world.

## 1. Introduction

**Figure 1.**
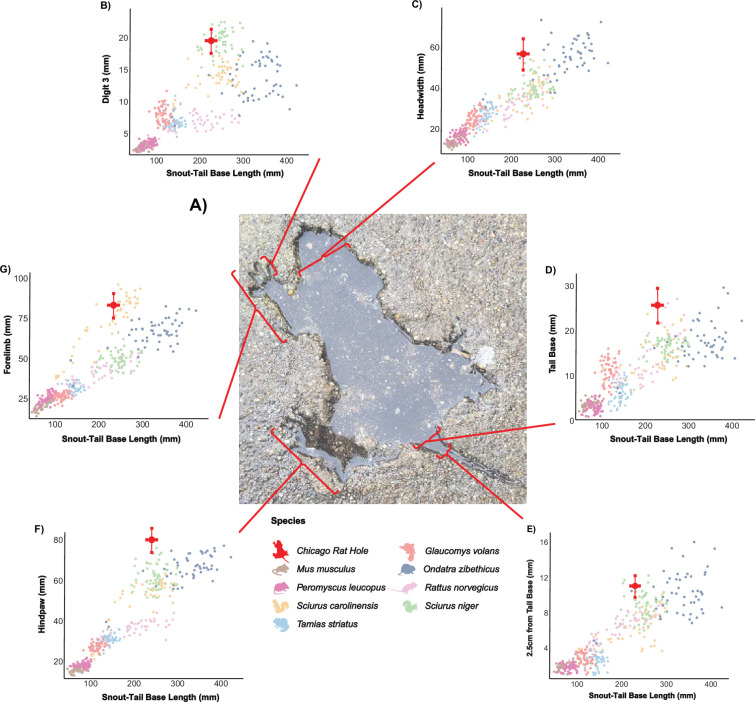
Comparative analysis of the ‘Chicago Rat Hole’ specimen (A), represented by the dark red dot in each graph. Lines extending from the dot indicate the standard deviation associated with measurement error across multiple photographs. In each plot, snout-to-tail length (STL) is shown on the *x*-axis, while a different morphological variable (B–G) is plotted on the y-axis using raw measurements. Bracketed lines link each bivariate plot to the specific reference measurement. Across all graphs, the ‘Chicago Rat Hole’ consistently overlaps with the Eastern grey squirrel (*Sciurus carolinensis*; yellow), fox squirrel (*Sciurus niger*; green) and muskrat (*Ondatra zibethicus*; dark blue). Photo of the ‘Chicago Rat Hole’ was taken by Winslow Dumaine and released under a Creative Commons Attribution-ShareAlike 4.0 International License (CC BY-SA 4.0). The Wikimedia Foundation has confirmed permission via Volunteer Response Team ticket no. 2024011910008464.

The ‘Chicago Rat Hole’ (figure 1), located originally in the Roscoe Village neighbourhood in Chicago, Illinois, became a viral sensation after comedian and writer Winslow Dumaine tweeted about its presence in early 2024 [1–3]. This seemingly ordinary sidewalk hole quickly inspired a wave of visitors who made pilgrimages to the site, leaving offerings like coins, flowers, figurines and even medication [1,4]. Though the hole had existed for 20–30 years, it rapidly became a local icon, with a nearby softball team adopting the rat as its mascot. The hole was named ‘Splatatouille’ after a public naming contest, and despite being removed in April 2024, it remains a piece of Chicago folklore, marked by a plaque from the Riot Fest Historical Society [1–3]. Following its removal by the Chicago Department of Transportation in April 2024, the slab was transported to the City Hall-County Building, where it currently resides.

From a scientific perspective, the ‘Chicago Rat Hole’ represents a remarkable modern full-body impression [5]. Full-body impressions are shallow, highly detailed imprints made by animals while alive or peri-mortem, typically preserved in soft sediment. In this instance, the tracemaker itself was not preserved but instead left behind a finely detailed impression in the surrounding substrate, offering a rare neoichnological analogue for interpreting trace-producing behaviours [6]. With such remarkable preservation, the ‘Chicago Rat Hole’ was named based on the assumption that the original imprint-maker was an unfortunate brown rat (*Rattus norvegicus*), a species quite common in urban centres, that met its end soon after a fresh layer of sidewalk concrete was poured [1–4]. While the taxonomic attribution of this imprint is not without validity, considering the commonality of brown rats in the region where the imprint was cast and the overall rodent-like appearance of the impression [4], no alternative hypothesis testing was conducted to rule out other possibilities. Therefore, this attribution was not based on the proper application of the scientific method, potentially obscuring both the identification of the true tracemaker and the historical context that led to its deposition.

In this study, we test the hypothesis that the ‘Chicago Rat Hole’ is truly attributable to a brown rat, or whether another sympatric species is a more likely tracemaker. To test this, we compare a series of linear measurements derived from anatomically identifiable landmarks on photographs of the ‘Chicago Rat Hole’ (figure 1) and compare them to measurements collected from museum specimens of commonly observed tetrapod taxa in the Chicago area. These data not only reveal the identity of the tracemaker behind the ‘Chicago Rat Hole’ but also serve as a neontological exemplar of the challenges in assigning taxonomic classification to full body impressions [5]. Additionally, they provide a tool for promoting the excitement of scientific exploration by making the scientific method accessible, engaging and relevant to a modern public audience.

## 2. Material and methods

### (a) Potential tracemakers

To identify the potential tracemaker responsible for the ‘Chicago Rat Hole’, we first isolated tetrapod species occurring within Chicago, Illinois, using iNaturalist [7,8]. Entire lineages could be dismissed based on readily distinguishable characteristics (e.g. the presence of four morphologically similar limbs excludes birds and snakes, while the presence of a tail excludes virtually all anurans and turtles), leaving mammals as the most likely candidates for the impression. iNaturalist [7,8] currently lists 37 mammalian species observed within Chicago. Most species can be further ruled out based on either rarity of occurrence (e.g. feral hogs and Mongolian jirds) or distinguishing characteristics such as the imprint’s lack of wings (i.e. bats), size (e.g. deer, coyotes, beavers, domestic dogs, cats and groundhogs) and the presence of a long tail (i.e. lagomorphs and moles).

The most distinguishing feature of the ‘Chicago Rat Hole’ for broad-scale taxonomic identification is the number of digits. The impression clearly shows five clawed digits on the hindpaw, while the forepaw displays only four. This pattern is consistent with rodents in which the pollex is greatly reduced compared to the other digits [9,10]. Based on these observations, the primary potential tracemakers among local species include the brown rat (*R. norvegicus*), house mouse (*Mus musculus*), Eastern grey squirrel (*Sciurus carolinensis*), Eastern chipmunk (*Tamias striatus*), muskrat (*Ondatra zibethicus*), white-footed mouse (*Peromyscus leucopus*), fox squirrel (*Sciurus niger*) and Southern flying squirrel (*Glaucomys volans*).

### (b) Anatomical measurements

To identify the taxonomic identity of the ‘Chicago Rat Hole,’ we first isolated clear anatomical landmarks for use in linear measurements. The animal’s positioning during deposition limited measurements to snout-to-tail base length (STL), forelimb length (FL), third digit length (D3L), hindpaw length (HPL), head width (HW), tail base width (TBW) and 2.5 cm from tail base width (2.5 TBW) (figure 1). As we were unable to collect our own photographs or three-dimensional reconstructions of the ‘Chicago Rat Hole’ prior to the onset of this study, we had to rely on images available on the internet. As almost all these images were captured from imperfect views for measurement purposes, we attempted to account for such measurement error by searching for all available images of the ‘Chicago Rat Hole’. We included images that clearly displayed all relevant landmarks and had a visible scale (e.g. coins), resulting in 25 suitable images. We distributed these images to co-authors (M.C.G., G.N.G., C.J.L.), who each collected the seven measurements from every suitable image using ImageJ, resulting in a total of 75 ‘virtual specimens’ used for subsequent statistical testing. We also gathered the same measurements from prepared museum skins (approx. 50 individuals) of each target species. To account for the unknown age of the ‘Chicago Rat Hole’ tracemaker, we specifically included specimens representing a broad range of body sizes to avoid prematurely dismissing the possibility of the tracemaker being a juvenile or particularly large adult. Individuals from both sexes were also tested, as the sex of the tracemarker was not known.

### (c) Statistical testing

To determine the taxonomic identity of the ‘Chicago Rat Hole,’ we conducted a series of statistical analyses using log-transformed morphometric data. All analyses were performed in R [11], using the MASS package [12] for discriminant function analysis (DFA), the Hotelling package [13] for Hotelling’s T²-tests and the stats package [14] for *t*-tests and principal component analysis (PCA).

We performed independent two-sample *t*-tests to compare the ‘Chicago Rat Hole’ specimen against each known species for every morphological variable. These tests assessed whether the unknown specimen differed significantly in raw trait values from individual species. No interspecific comparisons among the known species were conducted, as the objective was not to evaluate species-level differences within the dataset. Accordingly, no *post-hoc* corrections were applied. In addition, we plotted linear regressions between each log-transformed morphological variable and log-transformed STL. These regressions served an illustrative purpose rather than a statistical test; their primary goal was to visualize absolute size similarities between the unknown specimen and potential matches.

We performed PCA on the log-transformed morphometric dataset to identify major axes of morphological variation and reduce dimensionality. This approach allowed us to visualize how traits co-varied across specimens and where the ‘Chicago Rat Hole’ specimen fell within this multivariate space. PCA provided a useful framework for intuitively assessing its morphological similarity or divergence relative to known taxa.

To further assess inter-species differences, we conducted a DFA using all log-transformed variables to determine whether the ‘Chicago Rat Hole’ occupied a distinct region of the morphospace. All specimens were included in a single DFA to generate discriminant axes that maximally separated known species based on morphology. We then performed *post-hoc* pairwise comparisons between the ‘Chicago Rat Hole’ and each known species using Hotelling’s T²-tests. No pairwise comparisons were made among the known species. To assess how the model reclassifies the ‘Chicago Rat Hole’, a dropped group analysis was conducted, where the model was trained without data from the ‘Chicago Rat Hole’ and then predicted the species using the withheld data. To assess individualized differences between the ‘Chicago Rat Hole’ and the eight selected species, another Hotelling’s T^2^ test was used to determine the degree of difference of centroid means between the ‘Chicago Rat Hole’ and the other comparative local species. A larger T^2^ value indicates larger differences between centroid means.

## 3. Results

Utilizing log-transformed variables and *t*-tests, Eastern grey squirrels showed no significant difference from the ‘Chicago Rat Hole’ specimen in STL (*p* = 0.599). Muskrats were not significantly different in HW (*p* = 0.438) and 2.5 TBW (*p* = 0.476). Fox squirrels also showed no significant differences in D3L (*p* = 0.781) and 2.5 TBW (*p* = 0.096). All other species and variables differed significantly from the ‘Chicago Rat Hole’ specimen (*p* < 0.05) (figure 1 and electronic supplementary material, table S1).

**Figure 2.**
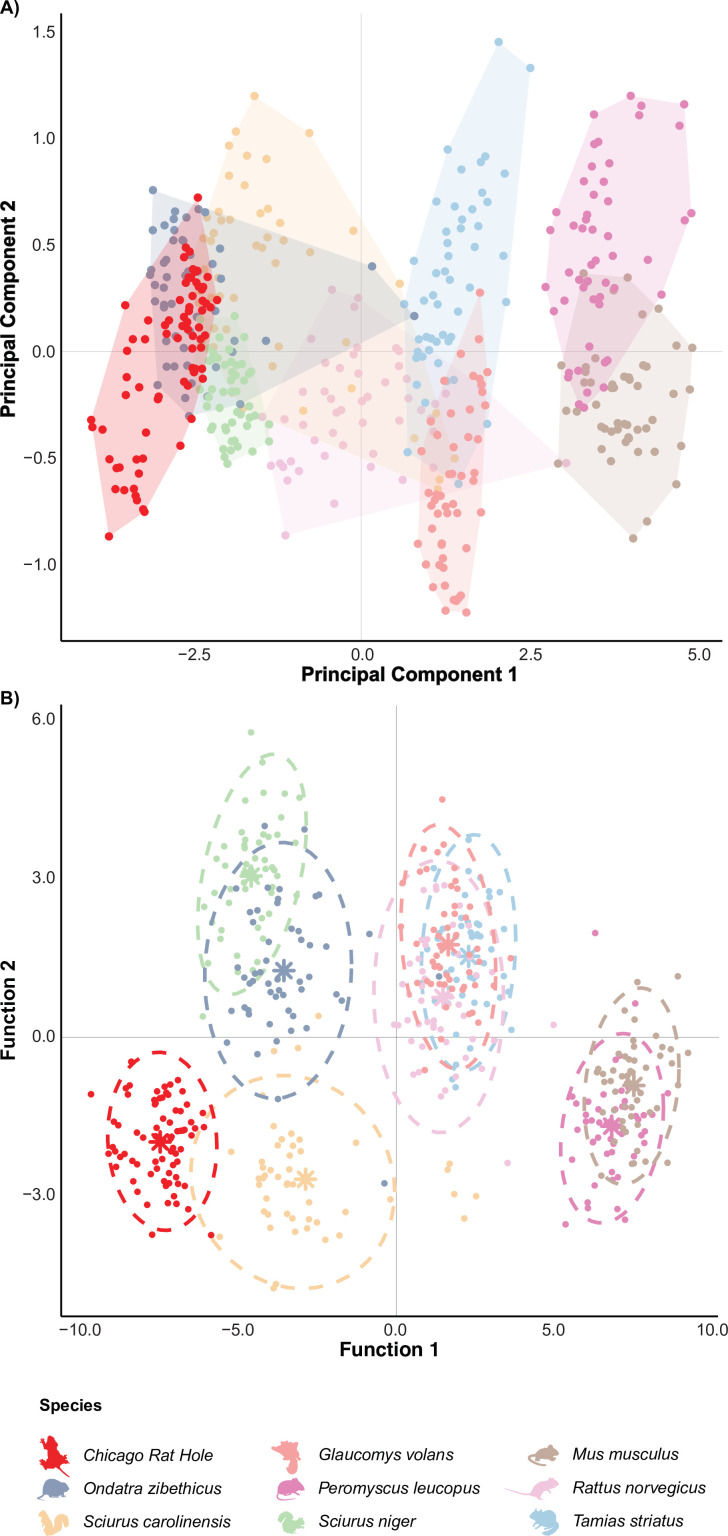
Results of the principal component analysis (A) and discriminant function analysis (B) performed on the full dataset, using log-transformed morphological measurements. The morphospace is plotted along the first two principal components (PC1 and PC2), which together capture 92.4% of the total variance (A) and first two linear discriminant functions, which together capture 86.4% of the between group variance (B). Points represent individual observations projected on the first two principal components (A) and linear discriminants (B).

Results from the PCA revealed strong morphological separation among species, with the first two principal components explaining 92.4% of the total variance (PC1 = 89.1%, PC2 = 3.3%; figure 2). Principal component 1 reflected overall size variation, with all variables contributing negatively and relatively equally (loadings ranging from −0.37 to −0.39). As such, species positioned on the left side of the morphospace tend to be larger across all measured traits, while those on the right are relatively smaller. Principal component 2 captured shape variation, particularly contrasting FL (loading = 0.50) and tail base width (loading = −0.74). Species located higher along principal component 2 exhibit relatively elongated forelimbs and narrower tail bases, whereas those lower on principal component 2 have broader tail bases and shorter forelimbs. The ‘Chicago Rat Hole’ falls in the centre-left region of the morphospace, clustering most closely with muskrats, Eastern grey squirrels and fox squirrels (electronic supplementary material, table S2).

**Figure 3.**
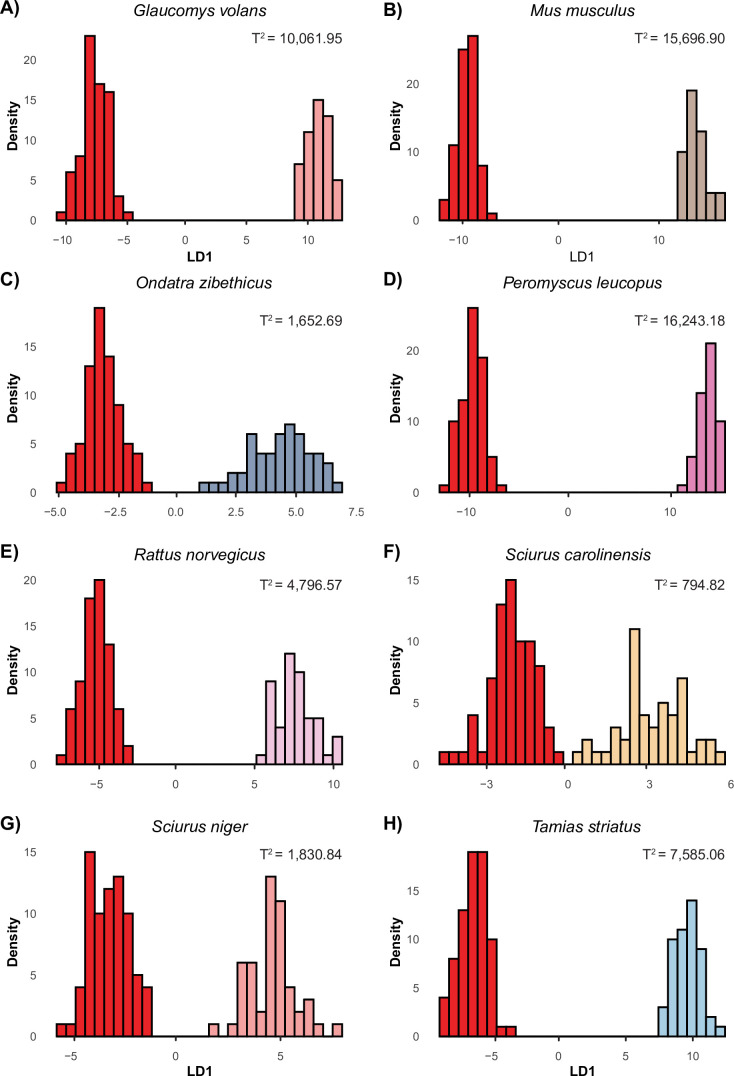
Density plots showing the first linear discriminant function (LD1) comparing the ‘Chicago Rat Hole’ (dark red) to each comparative species (A–H). All Hotelling’s T^2^-values are statistically significant (all *p-*values < 0.001).

Results from the DFA (figure 2) further confirmed strong morphological separation among species. Seven discriminant functions were produced, with function 1 accounting for 75.68% of the variance (eigenvalue = 1497.72; canonical r = 38.70), and function 2 accounting for 10.73% of the variance (eigenvalue = 212.31; canonical r = 14.57). Wilks’ lambda was significant (Λ = 1.74 × 10^-13^; d.f. = 7; χ² = 13838.51; *p* < 0.001), demonstrating significant differences between the eight species and the “Chicago Rat Hole’ (electronic supplementary material, tables S34). Classification accuracy was 93.54% (cross-validation = 92.92%). Further evaluating the loadings, Hotelling’s T-squared distribution showed the ‘Chicago Rat Hole’ centroid was closest in distribution to Eastern grey squirrels (T^2^ = 794.82; *p* < 0.001), muskrats (T^2^ = 1,652.69 l; *p* < 0.001) and fox squirrels (T^2^ = 1,830.84; *p* < 0.001) (figure 3 and electronic supplementary material, table S5). The dropped group analysis reclassified the measurements of the ‘Chicago Rat Hole’ into the genus *Sciurus*, with 50.67% classified as Eastern grey squirrels and 48.00% as fox squirrels. One measurement (1.33%) was classified as muskrats (electronic supplementary material, table S6).

## 4. Discussion

Taken together, these results suggest that the ‘Chicago Rat Hole’ most closely resembles the Eastern grey squirrel, fox squirrel or muskrat. Although the specimen was statistically distinct from all known species in multivariate space (figure 3 and electronic supplementary material, table S5), select univariate comparisons revealed no significant differences in key traits—specifically, STL in the Eastern grey squirrel, HW and 2.5 TBW in the muskrat, and D3L and 2.5 TBW in the fox squirrel (electronic supplementary material, table S1). These findings are further supported by the PCA, where the specimen occupies a central position overlapping with the same three species (figure 2A), the DFA (figure 2B), which yielded the lowest Hotelling’s T² values in comparisons with them (figure 3), and the dropped group analysis, which reclassified the ‘Chicago Rat Hole’ mostly into the genus *Sciurus* (electronic supplementary material, table S6).

Our analyses offer little support for the hypothesis that the ‘Chicago Rat Hole’ was made by a brown rat. The specimen’s relatively elongated forelimbs, third digits and hindpaws exceeded the measurement ranges observed in the brown rat. Instead, the data point to a closer resemblance with large-bodied rodents that exhibit generalized limb and tail proportions. While Eastern grey squirrels, fox squirrels and muskrats all fall within this morphological space, the measurements derived from the impression do not allow for definitive species-level identification.

Given the relative abundance of Eastern grey squirrels in the Chicago area compared to the much rarer fox squirrel or muskrat [8,15], incorporating density-based likelihoods into our interpretation would likely favour Eastern grey squirrels as the most probable identity. Still, our data strongly suggest that the ‘Chicago Rat Hole’ does not reflect a murid tracemaker. We therefore propose that the specimen be rechristened the ‘Windy City Sidewalk Squirrel’—a name more fitting of its likely origins and more aligned with the evidence at hand.

This study is not the first to suggest that the ‘Chicago Rat Hole’ may have been created by a squirrel [4]. In an interview with NBC Chicago, Dr Seth Magle, Lincoln Park Zoo’s Director of the Urban Wildlife Institute, proposed several factors that better align the maker of the impression with a squirrel. For instance, concrete is typically wet during the day, coinciding with diurnal squirrel activity [16,17]. Brown rats, being nocturnal [18], are far less likely to encounter wet concrete at an appropriate time to leave such an impression. Furthermore, Magle [4] noted the absence of other tracks leading to the impression, suggesting that the maker landed from above. There is no evidence in the impression to suggest that the unfortunate tracemaker walked away after its fall. While it is possible that a brown rat was dropped by a bird of prey [19], it is far more likely that a squirrel, despite their agility, misjudged a leap or slipped from a branch and fell, leaving the impression [4]. This theory is supported by reports from longtime residents in the area, who noted the former presence of a tree near the location of the impression [4]. Falls from heights are quite common for tree squirrels, as evidenced by the high incidence of skeletal markers attributable to fall-related injuries [20]. This risk is particularly pronounced for Eastern grey squirrels in urban centres, which are approximately 4.5 times more likely to have healed injuries compared to their rural counterparts [20].

The best argument against the ‘Chicago Rat Hole’ being created by a squirrel is the lack of the characteristic bushy-tail in the impression. Observing hair impressions in a full body impression is inherently challenging due to the delicate and ephemeral nature of hair compared to more robust biological structures like bones or scales. Hair lacks the rigidity to create deep, well-defined impressions in substrates, making it less likely to leave discernible traces under typical depositional conditions [21,22]. Sidewalk concrete, while capable of capturing impressions due to its initial plasticity, is not an ideal medium for preserving detailed biological features like hair. Concrete typically sets too quickly and lacks the fine-grained texture required to capture intricate details. Fine-grained sediments, such as silts, clays and very fine sand, are the most effective at preserving delicate impressions like hair [23]. These sediments provide a soft, pliable surface that can capture intricate details of an organism’s integuments before hardening into rock [23–25]. Taken together, it would actually be quite surprising if a bushy tail had been preserved, and this certainly does not provide sufficient evidence to argue against the ‘Chicago Rat Hole’ being attributable to a squirrel.

The preservation quality of the ‘Chicago Rat Hole’ underscores broader challenges in assigning taxonomic identity to full body impressions, even under near-ideal conditions [5,24,26]. Despite its distinct outline and clearly defined anatomical regions, and the unusual ability to narrow the tracemaker to one of eight plausible species, we were still only able to extract seven measurable traits and could not resolve its identity beyond the generic level. This outcome, alongside well-documented cases of misclassification in the literature [27–30], reinforces the inherent uncertainty of taxonomic inference from impressions alone. Compounding these challenges are the mechanics of imprint formation, which can distort original morphology [31]. As the tracemaker displaces sediment, lateral spreading can enlarge the impression [32], potentially accounting for morphological overlap between species such as the Eastern grey squirrel and the larger-bodied fox squirrel. Moreover, accurately reconstructing the depositional context of a trace impression is often difficult, even in neoichnological examples. For example, in the case of the ‘Chicago Rat Hole,’ it is unclear whether the animal entered the substrate vertically or at an angle—an oblique impact could result in foreshortening of the body, especially if the head contacted the surface before the hindlimbs, thereby distorting anatomical proportions and obscuring key measurements. That even a well-preserved and contextually constrained specimen like the ‘Chicago Rat Hole’ resists definitive identification illustrates the limitations of taxonomic resolution using impressions alone. In the absence of direct association between trace and tracemaker [33], caution is warranted when attributing full body impressions to specific taxa. These difficulties are only amplified in palaeontological contexts, where the possible age of a trace fossil may span millions of years, faunal composition is often incompletely known, and original surface morphology may be obscured or lost (e.g. in undertracks), further complicating taxonomic assignment [34].

### (a) Implications for scientific engagement

Throughout this article, we have employed traditional morphometric techniques to explore the taxonomic identity of the ‘Chicago Rat Hole’. While we, the authors, acknowledge the lighthearted nature of this exercise, the ‘Chicago Rat Hole’ nonetheless presents meaningful opportunities to promote scientific engagement. The ‘Chicago Rat Hole’ garnered widespread attention on social media [1–3], prompting the public to consider questions such as which species created the imprint and the depositional factors involved in its formation [4]. Such curiosity is fundamental to the scientific process. Even though the popular conclusion that the ‘Chicago Rat Hole’ was created by a brown rat is likely incorrect, the public’s reasoning—based on the imprint’s overall shape and the abundance of brown rats in Chicago [4]—demonstrates an impressive use of inductive logic that the scientific community should encourage. Moreover, the ‘Chicago Rat Hole’ fosters connections between urban residents and their non-human neighbours, illustrating that cities are thriving ecosystems where numerous species adapt and survive [35]. As Magle [4] aptly stated, ‘I think what it reminds us is that, no matter how deep in the city you live, we all have this deep tie, this need to be connected to nature and other species’.

Given the public’s interest, adapting similar analyses into outreach events [36] appears particularly promising for introducing students to concepts such as urban ecology, ichnofossils and morphometric techniques. We hope this work—in spite of (or perhaps more specifically, because of) its inherent frivolity—resonates with both the public and the scientific community, demonstrating that scientific inquiry does not need to be confined to laboratories or burdened with impenetrable jargon. At its core, science simply requires curiosity and a commitment to understanding the natural world around us.

## Data Availability

All data are made available in Supplemental Data 1. Supplementary material is available online [37].

## References

[1] Filbin P. 2025 Chicago’s Rat Hole, 1 Year Later: Where Is It Now? Block Club Chic. See https://blockclubchicago.org/2025/01/10/chicagos-rat-hole-1-year-later-where-is-it-now/ (accessed 12 June 2025).

[2] Coffman R. 2024 Chicago Rat Hole: Silly, Sacrilege, or Sacred? Chicago History Museum. See https://www.chicagohistory.org/chicago-rat-hole-silly-sacrilege-or-sacred/ (accessed 12 June 2025).

[3] Cao S. 2024 Long live the chicago rat hole. The Atlantic. See https://www.theatlantic.com/family/archive/2024/01/chicago-rat-hole-community-monument-meaning-absurd/677248/ (accessed 12 June 2025).

[4] NBC Chicago New. 2024 Zoo expert reveals 3 reasons why Chicago’s ‘rat hole’ might not be a rat. NBC Chic. See https://www.nbcchicago.com/news/local/zoo-expert-reveals-3-reasons-why-chicagos-rat-hole-might-not-be-a-rat/3329043/ (accessed 23 November 2024).

[5] Benner JS, Knecht RJ. 2025 Full-body impressions: a category of trace fossils unique to exceptionally preserved bedding planes and indicators of true substrates. Geol. Soc. Lond. Spec. Publ. **556**, SP556–2024. (10.1144/SP556-2024-87)

[6] Lallensack JN, Leonardi G, Falkingham PL. 2025 Glossary of fossil tetrapod tracks. Palaeontol. Electron. **28**, 1–105. (10.26879/1389)

[7] Di Cecco GJ, Barve V, Belitz MW, Stucky BJ, Guralnick RP, Hurlbert AH. 2021 Observing the observers: how participants contribute data to inaturalist and implications for biodiversity science. BioScience **71**, 1179–1188. (10.1093/biosci/biab093)

[8] Matheson CA. 2014 iNaturalist. Ref. Rev. **28**, 36–38. (10.1108/RR-07-2014-0203)

[9] Samuels JX, Van Valkenburgh B. 2008 Skeletal indicators of locomotor adaptations in living and extinct rodents. J. Morphol. **269**, 1387–1411. (10.1002/jmor.10662) 18777567

[10] Brown TG. 1911 The intrinsic factors in the act of progression in the mammal. Proc. R. Soc. Lond. B **84**, 308–319. (10.1098/rspb.1911.0077)

[11] R Core Team. 2021 R: a language and environment for statistical computing. Vienna, Austria: R Foundation for Statistical Computing. See https://www.R-project.org/.

[12] Ripley B, Venables B, Bates DM, Hornik K, Gebhardt A, Firth D, Ripley MB. 2013 Package ‘mass.’ Cran R **538**, 822.

[13] Curran J, Curran MJM. 2017 Package ‘Hotelling.’ R Package Version 1–0.

[14] Crawley MJ. 2014 Statistics: an introduction using r. London, UK: John Wiley & Sons. (10.1002/9781119941750)

[15] van der Merwe M, Brown JS, Jackson WM. 2005 The coexistence of fox (Sciurus niger) and gray (S. caroliniensis) squirrels in the Chicago metropolitan area. Urban Ecosyst. **8**, 335–347. (10.1007/s11252-005-4865-9)

[16] Thompson DC. 1977 Diurnal and seasonal activity of the grey squirrel (Sciurus carolinensis). Can. J. Zool. **55**, 1185–1189. (10.1139/z77-153)

[17] Wassmer T, Refinetti R. 2019 Individual daily and seasonal activity patterns in fox squirrels (Sciurus niger) quantified by temperature-sensitive data loggers. Front. Ecol. Evol. **7**. (10.3389/fevo.2019.00179)

[18] Byers KA, Lee MJ, Patrick DM, Himsworth CG. 2019 Rats about town: a systematic review of rat movement in urban ecosystems. Front. Ecol. Evol. **7**. (10.3389/fevo.2019.00013)

[19] Switzer PV. 1999 Avian prey-dropping behavior. I. The effects of prey characteristics and prey loss. Behav. Ecol. **10**, 213–219. (10.1093/beheco/10.3.213)

[20] Moncrief ND, Hightower L, Mead AJ, Ivanov K. 2022 Prevalence and location of survivable skeletal injuries in two North American tree squirrels (Sciurus). J. Mammal. **103**, 663–671. (10.1093/jmammal/gyab131)

[21] Butterfield NJ. 1990 Organic preservation of non-mineralizing organisms and the taphonomy of the Burgess Shale. Paleobiology **16**, 272–286. (10.1017/s0094837300009994)

[22] Allison PA, Briggs DEG. 1993 Exceptional fossil record: distribution of soft-tissue preservation through the Phanerozoic. Geology **21**, 527. (10.1130/0091-7613(1993)0212.3.co;2)

[23] Gaines RR, Hammarlund EU, Hou X, Qi C, Gabbott SE, Zhao Y, Peng J, Canfield DE. 2012 Mechanism for Burgess Shale-type preservation. Proc. Natl Acad. Sci. USA **109**, 5180–5184. (10.1073/pnas.1111784109) 22392974 PMC3325652

[24] Marchetti L *et al*. 2019 Defining the morphological quality of fossil footprints. Problems and principles of preservation in tetrapod ichnology with examples from the Palaeozoic to the present. Earth Sci. Rev. **193**, 109–145. (10.1016/j.earscirev.2019.04.008)

[25] Nigro M, MacKenzie L, SUNY Geneseo. 2017 Experimental taphonomy: the effects of sediment grain size on microbial and mineral film growth on tissues. In GSA Annual Meeting, Seattle, WA. Boulder, CO: The Geological Society of America. (10.1130/abs/2017AM-305333)

[26] Sarjeant WAS. 1975 Fossil tracks and impressions of vertebrates. In The study of trace fossils a synthesis of principles, problems, and procedures in ichnology (ed. R Frey), pp. 283–324. Berlin, Germany: Springer Berlin Heidelberg. (10.1007/978-3-642-65923-2_14)

[27] Bowden AJ, Tresise GR, Simkiss W. 2010 Chirotherium, the Liverpool footprint hunters and their interpretation of the Middle Trias environment. In Dinosaurs and other extinct saurians: a historical perspective (eds E Buffetaut, D Naish, DM Martill), pp. 209–228, vol. **343**. (10.1144/SP343.12)

[28] Padian K, Olsen PE. 1984 The fossil trackway Pteraichnus: not pterosaurian, but crocodilian. J. Paleontol **58**, 178–184. https://www.jstor.org/stable/1304743

[29] Lockley MG, Logue TJ, Moratalla JJ, Hunt AP, Schultz RJ, Robinson JW. 1995 The fossil trackway Pteraichnus is pterosaurian, not crocodilian: implications for the global distribution of pterosaur tracks. Ichnos **4**, 7–20. (10.1080/10420949509380110)

[30] Jensen S. 2003 The Proterozoic and earliest Cambrian trace fossil record; patterns, problems and perspectives. Integr. Comp. Biol. **43**, 219–228. (10.1093/icb/43.1.219) 21680425

[31] Gatesy SM, Falkingham PL. 2017 Neither bones nor feet: track morphological variation and ‘preservation quality'. J. Vertebr. Paleontol. **37**, e1314298. (10.1080/02724634.2017.1314298)

[32] Gatesy S. 2003 Direct and indirect track features: what sediment did a dinosaur touch? Ichnos **10**, 91–98. (10.1080/10420940390255484)

[33] Nyakatura JA *et al*. 2019 Reverse-engineering the locomotion of a stem amniote. Nature **565**, 351–355. (10.1038/s41586-018-0851-2) 30651613

[34] Hedrick BP. 2023 Dots on a screen: the past, present, and future of morphometrics in the study of nonavian dinosaurs. Anat. Rec. **306**, 1896–1917. (10.1002/ar.25183) 36922704

[35] Savard JPL, Clergeau P, Mennechez G. 2000 Biodiversity concepts and urban ecosystems. Landsc. Urban Plan. **48**, 131–142. (10.1016/s0169-2046(00)00037-2)

[36] Young MW *et al*. 2025 ’Are you stronger than a lemur?’ An effective, interactive STEM outreach program for increasing anatomical and biomechanical knowledge across diverse populations. Anat. Sci. Educ. (10.1002/ase.70012) 40104959

[37] Granatosky MC, Guilhon GN, Chernik ND, Kantonis SJ, Lee CJ, Dickinson E. 2025 Supplementary material from: Rodent indent not self-evident: a case of mistaken identity of the ‘Chicago Rat Hole'. Figshare. (10.6084/m9.figshare.c.8079966) PMC12521980 41089057

